# Prospective study on planned biliary stent placement to treat small common bile duct stones

**DOI:** 10.1002/jgh3.13040

**Published:** 2024-02-23

**Authors:** Shuzo Terada, Shinya Kawaguchi, Eiji Nakatani, Atsuko Inagawa, Tomoki Hikichi, Shodai Takeda, Yuya Ishiguro, Hirotaka Kashima, Taiyo Hirata, Shinya Ikeda, Kazuhisa Asahara, Tatsunori Satoh, Yuichi Masui, Masanori Matsuda, Ryosuke Itai, Asami Kawai, Shinya Endo, Takafumi Kurokami, Naofumi Shirane, Kazuya Ohno

**Affiliations:** ^1^ Department of Gastroenterology Shizuoka General Hospital Shizuoka Japan; ^2^ Graduate School of Public Health (Medical Statistics) Shizuoka Graduate University of Public Health Shizuoka Japan

**Keywords:** choledocholithiasis, endoscopic sphincterotomy, recurrence, stents, stone clearance

## Abstract

**Aims:**

Small common bile duct stones are known to occasionally clear spontaneously. This study aimed to prospectively assess the role of biliary stent placement in promoting the spontaneous clearance of small common bile duct stones.

**Methods and Results:**

We analyzed patients presenting with common bile duct stones of ≤5 mm diameter between June 2020 and May 2022. The exclusion criteria included asymptomatic patients, biliary pancreatitis, altered gastrointestinal anatomy, bile duct strictures (malignant or benign), and a history of EST. The biliary stents were inserted without stone removal. Stone clearance was assessed using endoscopic ultrasonography or endoscopic retrograde cholangiopancreatography after 3 months. Our primary endpoint was the clearance rate of common bile duct stones over 6 months, targeting a lower limit for the 95% confidence interval (CI) exceeding 25%. Of the 32 enrolled patients, 18 (56.3%; 95% CI: 37.7–73.6%) exhibited stone clearance. Early complications occurred in 11 patients (34.4%), totaling 12 incidents: acute cholecystitis in four, acute pancreatitis in three, biliary pain in three, and cholangitis in two patients. No severe complications occurred. Six (18.8%) patients experienced asymptomatic stent migration. Following stone clearance, four (12.5%) patients experienced stone recurrence, with an average duration of 256 ± 164 days.

**Conclusion:**

Biliary stenting appeared to effectively promote the clearance of small common bile duct stones in approximately half of the patients. However, the potential complications and risks of stone recurrence warrant close monitoring.

This trial was registered in the Japan Registry of Clinical Trials (jRCT1042200020).

## Graphical abstract

This study aimed to prospectively assess the role of biliary stent placement in promoting the spontaneous clearance of common bile duct stones of ≤5 mm diameter. The biliary stents were inserted without stone removal. Of the 32 enrolled patients, 18 (56.3%; 95% CI: 37.7–73.6%) exhibited stone clearance over 6 months. Biliary stenting appeared to effectively promote the clearance of small common bile duct stones. However, the potential complications and risks of stone recurrence warrant close monitoring.

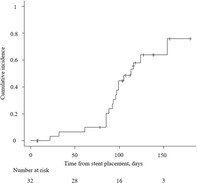

## Introduction

Endoscopic biliary stenting is the gold standard for acute cholangitis with choledocholithiasis owing to its minimally invasive approach.^1^ Temporary endoscopic placement of a biliary stent is also recommended for patients with unretrievable common bile duct stones who require biliary drainage.^2^ Biliary stents can fragment large stones, allowing them to pass spontaneously or making them easier to extract later, facilitating two‐stage stone removal.^3^, ^4^ Biliary stenting has been suggested to promote the safe passage of small choledocholithiasis.^3^, ^5^

Endoscopic biliary stenting is also being attempted to prevent biliary complications in the perioperative period of cholecystectomy for gallbladder stones after endoscopic sphincterotomy (EST) for common bile duct stones.^6^ Although cholecystectomy is recommended after endoscopic papillotomy for common bile duct stones concomitant with gallbladder stones, biliary complications have been reported to occur as frequently as approximately 20% while waiting for cholecystectomy.^7^ Therefore, early cholecystectomy is preferable, but laparoscopic cholecystectomy often requires a waiting period of several weeks for treatment due to patient preference, lack of surgeons, COVID‐19 pandemic, and other factors.^8^, ^9^

EST is the standard treatment for common bile duct stones owing to its safety and effectiveness.^2^, ^10^ However, EST is associated with several complications. Early complications include perforation and bleeding, and patients taking oral anti‐thrombotic drugs and those on hemodialysis have also been reported to be important risk factors for post‐EST bleeding.^10^, ^11^ Additionally, late complications such as the recurrence of bile duct stones, cholangitis, and cholecystitis that develop after EST due to the permanent loss of sphincter function are also a concern.^12^

Endoscopic biliary stenting for small common bile duct stones is a treatment that may avoid complications associated with EST by promoting stone clearance. However, biliary stenting for several months may cause complications, such as cholangitis and cholecystitis, due to prolapse or obstruction. This study aimed to prospectively collect cases of planned biliary stenting for small common bile duct stones and evaluate the efficacy and safety of this approach.

## Methods

### Study design, ethics, and trial registration

We conducted a prospective, single‐center, single‐arm cohort trial involving patients diagnosed with common bile duct stones measuring ≤5 mm in their shortest diameter. The patients underwent biliary stent placement between June 2020 and May 2022 at the Shizuoka General Hospital. The study was conducted in accordance with the guidelines of the Declaration of Helsinki and was approved by the ethics committee of Shizuoka General Hospital (SGHIRB#2020007). Written informed consent was obtained from all patients before their participation in the study. This trial was registered in the Japan Registry of Clinical Trials (jRCT) (jRCT1042200020).

### Trial population

We included eligible patients who had a biliary stent placement and were diagnosed with common bile duct stones measuring ≤5 mm in their shortest diameter. If multiple common bile duct stones were present, the largest stone with the shortest diameter was measured. The stone diameter was measured by cholangiography; however, if measuring the stone diameter by cholangiography was challenging, it was measured by other imaging techniques, such as endoscopic ultrasonography (EUS), magnetic resonance imaging, and computed tomography (CT). The exclusion criteria were as follows: (1) bile duct malignant tumor, (2) history of Billroth‐II reconstruction, Roux‐en‐Y reconstruction gastrectomy, cholangiojejunostomy, or pancreaticoduodenectomy, (3) benign biliary stricture, (4) history of EST, (5) no symptoms, (6) gallstone pancreatitis, (7) concerned doctor has recommended that the candidate be excluded, and (8) patients without cholangitis and with a common bile duct stone diameter of ≥6 mm by endoscopic retrograde cholangiopancreatography (ERCP) performed after obtaining consent. The study physician obtained informed consent from all eligible patients. For patients who required emergency biliary stent placement due to acute cholangitis, this study was explained within 4 weeks after biliary stent placement. For patients without cholangitis, this study was explained before ERCP.

### Procedure and follow‐up

Patients were administered 50 mg of diclofenac rectally before ERCP unless contraindicated, and intravenous flunitrazepam was administered for conscious sedation. The procedures were executed by expert trainers who had performed more than 400 ERCPs or by trainees under expert supervision. A side‐viewing duodenoscope (TJF290V or JF260V; Olympus Medical Systems, Tokyo, Japan; or ED‐580 T; FUJIFILM, Tokyo, Japan) was used for ERCP. A curvilinear array echoendoscope (GF‐UCT260) or an electronic radial echoendoscope (GF‐UE260) (both from Olympus Medical Systems) paired with an ultrasound system (EU‐ME2; Olympus Medical Systems) were used for EUS. The double‐pigtail stent, Medi‐Globe 7 Fr 8 cm (Medico's Hirata Incorporation, Osaka, Japan), was placed mainly in the transpapillary with the upper end in the left or right intrahepatic bile ducts, but other plastic stents were used at the discretion of the endoscopist. We considered the placement of a pancreatic stent to prevent post‐ERCP pancreatitis in patients requiring more than three pancreatography procedures or using the pancreatic duct guidewire method.

Cholecystectomy is recommended for patients with gallstones *in situ*. EUS or ERCP with intraductal ultrasonography was performed 12–26 weeks after stent placement to evaluate stone clearance. If the common bile duct stones remained, EST was performed to remove the stones. Common bile duct stone recurrence was confirmed using imaging. Adverse events were evaluated using laboratory tests and clinical symptoms during EUS or ERCP, on the day after ERCP, and 2–4 weeks later. One year after the initial ERCP, stone recurrence was assessed using EUS, CT, or telephone interviews.

### Endpoints and other definitions

The primary endpoint was the rate of common bile duct stone clearance within 6 months of biliary stent placement. This was estimated using the Clopper–Pearson method. The passage of common bile duct stones was confirmed using EUS or cholangiography with intraductal ultrasonography. EUS was the preferred method of stone evaluation. Cholangiography was performed for patients with residual bile duct stones or inadequate evaluation of bile duct stones on EUS. Additional evaluation with cholangiography using a balloon catheter was performed in patients with pneumobilia for whom stone evaluation would be inadequate. Patients who could be traced after 6 months without confirming clearance were still included in the denominator and not excluded from the analysis. The efficacy of this treatment method was confirmed when the lower limit of the 95% confidence interval exceeded 25%. The secondary endpoints were the period from stent placement to stone clearance, rate of stone recurrence, time to recurrence, rate of complications, and asymptomatic stent migration.

Acute pancreatitis was defined according to the Revised Atlanta classification,^13^ acute cholangitis and acute cholecystitis were defined according to the Tokyo Guidelines 2018,^14^, ^15^ and other adverse events were defined according to the American Society for Gastroenterological Endoscopy (ASGE) guidelines for ERCP complications.^16^ The Common Terminology Criteria for Adverse Events ver.4.0 was used to evaluate adverse events not listed in the ASGE guidelines. Early complications were defined as those occurring from the time of stent placement to within 30 days after stone clearance on EUS or ERCP, whereas late complications were defined as those occurring after 30 days.

### Statistical analysis

Based on the results of our unpublished study examining the rate of stone clearance after biliary stent placement, we assumed a stone clearance rate of 44%. The spontaneous stone clearance rate was reported to be 19.3–22.7%,^17^, ^18^, ^19^ while the threshold clearance rate was determined to be 25%. Hence, we estimated that approximately 36 patients would be needed, with alpha (one‐sided) set at 0.05 and beta at 0.2. We anticipated that 20% of the patients would be excluded from the analysis during the follow‐up period for various reasons, thus setting the target number of registered patients at 45.

Descriptive statistics were used to summarize the baseline characteristics and clinical outcomes of patients. Continuous variables were presented as mean ± standard deviation (SD) when normally distributed or as median (interquartile range) for non‐normally distributed data. Categorical variables were expressed as frequencies and percentages. Any comparisons between groups, if made, would employ the appropriate statistical tests based on the data type and distribution.

The primary endpoint, that is, the clearance rate of common bile duct stones, was presented as a percentage with a 95% CI. The lower limit of the 95% CI for this outcome was of particular interest, with a target of exceeding 25%. The efficacy analysis did not include patients who underwent ERCP with consent but had common bile duct stones >6 mm. To find a patient population demonstrating the efficacy of this treatment, we conducted an additional analysis using the absence of common bile duct stones up to 6 months after bile duct stenting as an outcome.

For all analyses, a two‐tailed *P*‐value of <0.05 was considered statistically significant. All statistical analyses were conducted using SAS software (version 9.4; SAS Institute Inc., Cary, NC, USA).

## Results

### Baseline characteristics

Due to challenges in obtaining consent, we could not attain the initially planned sample size; thus, there were 32 participants at the end of the study period. No patients were excluded during the follow‐up period. The baseline characteristics of the patients are shown in Table 1. The mean ± SD age was 67.0 ± 14.9 years and 14 patients (43.8%) were females. Among the clinical presentations, we noted 13 cases of cholangitis, 16 of biliary colic, 2 of abnormal liver function, and 1 of cholecystitis. Twelve patients underwent emergency ERCP. The median short diameter of the largest common bile duct stones was 3.1 mm.

**Table 1:** Baseline characteristics of the study patients

Variable	Category or unit and statistics	Patients (*n* = 32)
Age	Years, mean ± SD	67.0 ± 14.9
Sex	Females	14 (43.8%)
Clinical presentation	Cholangitis	13 (40.6%)
	Biliary colic	16 (50.0%)
	Abnormal liver function	2 (6.3%)
	Cholecystitis	1 (3.1%)
Status of the gallbladder	Gallbladder with stones *in situ*	23 (71.9%)
	Gallbladder without stones *in situ*	2 (6.3%)
	Previous cholecystectomy	7 (21.9%)
Long diameter of the largest stone	mm, median (range)	4.3 (2–9.3)
Short diameter of the largest stone	mm, median (range)	3.1 (2–5)
Diameter of the bile duct	mm, median (range)	7.3 (3–12)
Number of stones	Single	19 (59.4%)
	Multiple	13 (40.6%)
Use of antithrombotic agents	Yes	7 (21.9%)
Periampullary diverticulum	Yes	8 (25%)
Emergency ERCP	Yes	14 (43.8%)
Type of biliary plastic stent	7Fr pigtail	23 (71.9%)
	7Fr straight	3 (9.4%)
	5Fr straight	6 (18.8%)
Pancreatic stent	Yes	8 (25%)

ERCP, endoscopic retrograde cholangiopancreatography; SD, standard deviation.

### Clinical outcomes and complications

**Figure 1:**
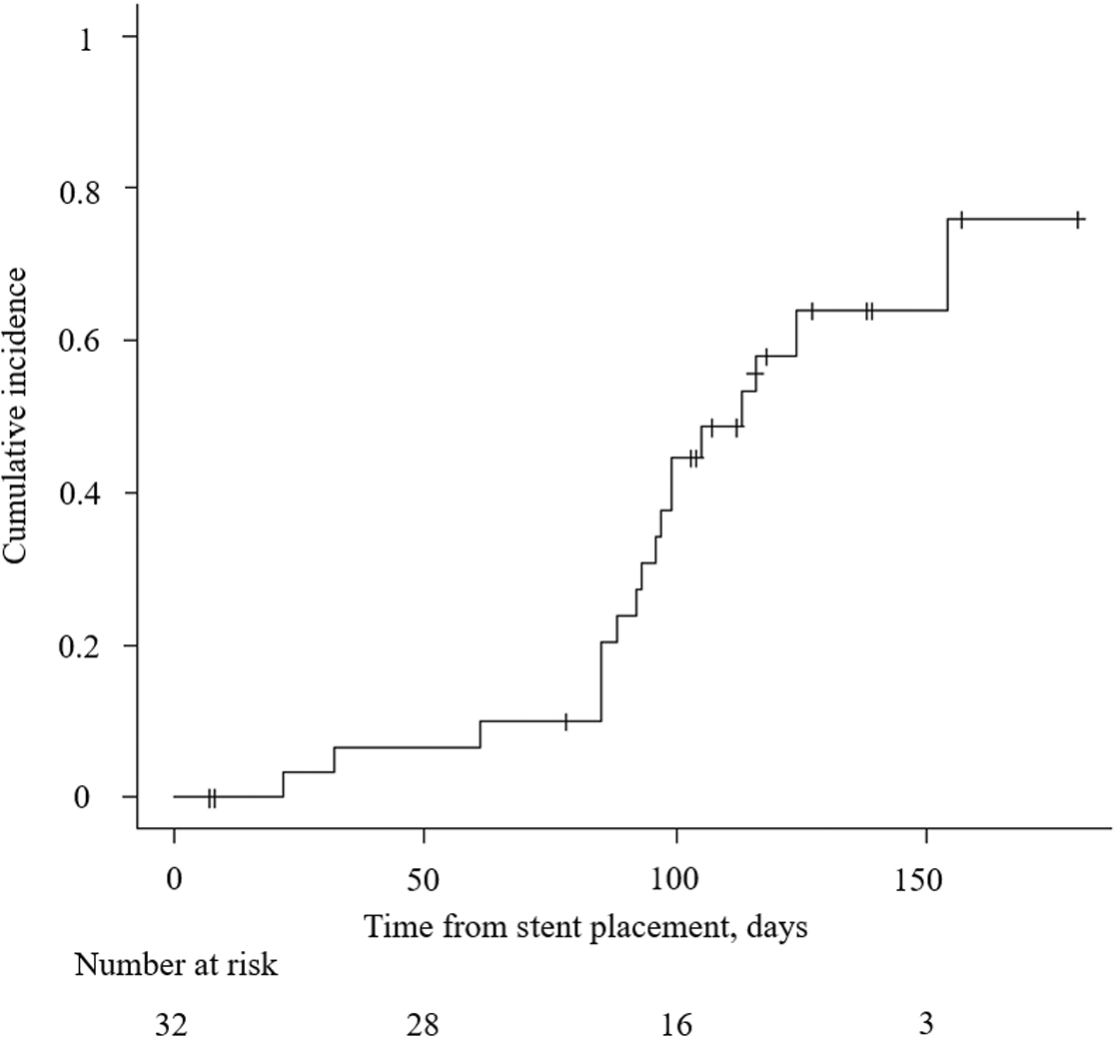
Cumulative incidence rate for stone clearance without endoscopic sphincterotomy.

The clinical outcomes and complications in the patients are shown in Table 2 and Figure 1. In 18 patients (56.3%), common bile duct stones resolved, with the lower limit of the 95% confidence interval exceeding 25%. On average, it took 97.2 ± 39.7 days from stent placement to stone clearance evaluation using imaging, with evaluations conducted for 16 patients using EUS and for the other 16 using ERCP. Of the 16 patients evaluated by ERCP, 9 patients were evaluated by EUS followed by ERCP. Of 23 patients with gallstones *in situ*, 21 (91.3%) underwent cholecystectomy. Among the 11 patients (34.4%) with early complications, 4 had acute cholecystitis, 3 had acute pancreatitis, 3 had biliary colic pain, and 2 had cholangitis. No severe complications occurred. Early complications occurred in 10 patients (31.3%) at the first ERCP and in 1 patient at the second ERCP. Asymptomatic stent migration occurred in six patients (18.8%). Stone recurrence occurred in four patients (12.5%) at an average of 256 ± 164 days after stone clearance.

**Table 2:** Clinical outcomes and complications

Variable	Category, or unit and statistics	Patients (*n* = 32)
Passage of common bile duct stones	*n*, % (95% CI)	18, 56.3% (37.7–73.6)
Duration from stent placement to the assessment of stone clearance	Days, mean ± SD	97.2 ± 39
Modality for assessment of stone clearance	EUS	16 (50%)
	ERCP	16 (50%)
Early complications†	Total	11 (34.4%)
	Mild PEP	3 (9.4%)
	Cholecystitis	4 (12.5%)
	Biliary colic	3 (9.4%)
	Cholangitis	2 (6.3%)
Asymptomatic migration of the stent	Yes	6 (18.8%)
Stone recurrence as a late complication^‡^	Yes	4 (12.5%)
Duration from stone clearance to stone recurrence	Days, mean ± SD	256 ± 164

^†^ Complications from stent placement to within 30 days after confirmation of stone clearance.

^‡^ Complications that occurred 30 days after stone clearance.

CI, confidence interval; EUS, endoscopic ultrasonography; ERCP, endoscopic retrograde cholangiopancreatography; PEP, post‐ERCP pancreatitis; SD, standard deviation.

### Additional analysis to determine factors contributing to the clearance of common bile duct stones

The baseline characteristics and outcomes for the stone‐clearance and no‐stone‐clearance groups are shown in Table 3. The common bile duct diameter was slightly smaller in the group with common bile duct stone clearance, showing a trend toward fewer early complications; however, no significant differences were observed. All six patients in whom the bile duct stent spontaneously migrated experienced common bile duct stone clearance (*P* = 0.024).

**Table 3:** Comparison of baseline characteristics and complications between the stone‐clearance group and no‐stone‐clearance group by univariate analysis

Variable	Category, or unit and statistics	Stone‐clearance group (*n* = 18)	No‐stone‐clearance group (*n* = 14)	*P* value
Age	Years, mean ± SD	68.2 ± 13.2	65.5 ± 17.2	0.62
Sex	Females	9 (50.0%)	5 (35.7%)	0.49
Clinical presentation	Cholangitis	8 (44.4%)	5 (35.7%)	0.5
	Biliary colic	8 (44.4%)	8 (57.1%)	
	Abnormal liver function	2 (11.1%)	0 (0.0%)	
	Cholecystitis	0 (0.0%)	1 (7.1%)	
Status of the gallbladder	Gallbladder with stones *in situ*	15 (83.3%)	8 (57.1%)	0.15
	Gallbladder without stones *in situ*	0 (0.0%)	2 (14.3%)	
	Previous cholecystectomy	3 (16.7%)	4 (28.6%)	
Maximum diameter of the largest stone	mm, median (range)	4.5 (2.0–9.3)	4.3 (3.0–8.0)	0.77
Minimum diameter of the largest stone	mm, median (range)	3.0 (2.0–5.0)	4.0 (3.0–5.0)	0.15
Diameter of the bile duct	mm, median (range)	6.0 (4.5–12.0)	8.3 (3.0–11.0)	0.2
Number of stones	Single	10 (55.6%)	9 (42.9%)	0.11
	Multiple	8 (61.5%)	5 (38.5%)	
Use of antithrombotic agents	Yes	4 (22.2%)	3 (21.4%)	>0.99
Periampullary diverticulum	Yes	7 (38.9%)	1 (7.1%)	0.053
Emergency ERCP	Yes	8 (44.4%)	6 (42.9%)	>0.99
Type of biliary plastic stent	7Fr pigtail	13 (72.2%)	10 (71.4%)	>0.999
	7Fr straight	2 (11.1%)	1 (7.1%)	
	5Fr straight	3 (16.7%)	3 (21.4%)	
Pancreatic stent	Yes	1 (7.1%)	7 (38.9%)	0.053
Duration from stent placement to the assessment of stone clearance	Days, median (range)	93.5 (21–153)	113 (6.0–179)	0.06
Early complications†	Total	3 (16.7%)	9 (64.3%)	0.094
	Mild PEP	1 (5.6%)	2 (14.3%)	
	Cholecystitis	2 (11.1%)	2 (14.3%)	
	Biliary colic	0 (0.0%)	3 (21.4%)	
	Cholangitis	0 (0.0%)	2 (14.3%)	
Asymptomatic migration of the stent	Yes	6 (33.3%)	0 (0.0%)	0.024
Migration of the stent	Yes	6 (33.3%)	2 (14.3%)	0.41
Stone recurrence as a late complication^‡^	Yes	3 (16.7%)	1 (7.1%)	>0.999

^†^ Complications from stent placement to within 30 days after confirmation of stone clearance.

^‡^ Complications that occurred 30 days after stone clearance.

CI, confidence interval; EUS, endoscopic ultrasonography; ERCP, endoscopic retrograde cholangiopancreatography; PEP, post‐ERCP pancreatitis; SD, standard deviation.

## Discussion

Although many reports have demonstrated the use of bile duct stents for difficult bile duct stones,^3^, ^4^ few studies have investigated the resolution of small stones without EST. Our prospective study revealed that 56% of common bile duct stones measuring ≤5 mm in diameter resolved after planned biliary stenting. As this was not a comparative study, it is unclear whether biliary stent placement contributed to the clearance of stones. However, this study showed a stone clearance rate surpassing that of previous reports, where untreated bile duct stones spontaneously passed at rates of 19.3–22.7%.^17^, ^18^, ^19^ This finding suggests that bile duct stenting is likely to be effective in improving stone clearance rates.

Three potential mechanisms may explain the clearance of bile duct stones after biliary stenting. First, stone fragmentation may result from mechanical friction with the stent.^5^ While it has been established that large stones diminish in size post‐stent placement,^3^ our study suggests that small stones either fragment or turn into sludge and are subsequently excreted through the papilla. The second mechanism is the spontaneous passage. Stones have been reported to clear from the papilla when bile excretion function is maintained, even without EST,^5^ and it is possible that the stones were cleared by passing through the side of the bile duct stent. The third possibility is that the stone accompanying the stent migrates into the duodenum and passes through the sphincter. In this study, common bile duct stones cleared in all six patients with asymptomatic migration of the stent, and spontaneous migration of the bile duct stent may be a sign of stone clearance.

However, we could not obtain the planned number of patients during the study period. This may be due to the difficulty in obtaining consent for participation in this study, which required two or more endoscopic examinations and increased the patient burden compared with the standard treatment method of performing EST at the time of the initial ERCP. Moreover, the probability of benefit from the study was not very high, as approximately half of the patients experienced stone clearance and preserved sphincter function even without EST.

The reported early complication rates for stone removal by EST range from 3% to 11.8%,^10^ so our study's rate of 34.4% is concerning. Despite the high rate of cholecystectomy in this study (91.3%), a median of 40.5 days post‐stent placement was observed, which coincided with a notable rate of acute cholecystitis (12.5%). Early cholecystectomy, as recommended by the European Society of Gastrointestinal Endoscopy guidelines,^2^ could have prevented cholecystitis. In addition, although 25% of the patients underwent pancreatic duct stenting, mild pancreatitis after ERCP occurred in 9.4% of cases.

The significance of preserving sphincter function is debatable;^20^ nonetheless, a randomized controlled trial of EPBD *versus* EST showed that preserving sphincter function may prevent stone recurrence in the long term.^12^ However, during the one‐year follow‐up observed in our study, stone recurrence occurred in 3 (16.7%) of the 18 patients whose stones cleared after planned stenting. One possible reason for stone recurrence is that EUS may miss small stones hidden behind the stent or in the hilar bile duct during bile duct stenting. There was one case of stone recurrence in a patient whose EUS showed biliary sludge; a biliary infection caused by the biliary sludge may have contributed to the recurrence of common bile duct stones.

Although planned biliary stenting effectively eliminated approximately half of the small stones, we must further probe its adverse events and long‐term outcomes. Therefore, a common bile duct stone without cholangitis, which is not usually indicated for biliary drainage, is unlikely to be a good indication for planned biliary stenting. Currently, planned biliary stenting is better reserved for patients with acute cholangitis who require emergency drainage and for whom early EST is contraindicated because of factors such as difficulty with anti‐thrombotic drug withdrawal or poor general condition.

This study has several limitations. The single‐center design restricted both the sample size and the broader applicability of our results. Second, this study was a single‐arm treatment that was not randomly assigned and contained a substantial risk of selection bias. Third, the stent placement period is necessary for further consideration. We set 3–6 months in consideration of recurrent biliary obstruction and cholecystitis during the waiting period; however, this may be useful even for a shorter period of placement. Finally, the study period may have been too short to evaluate late stone recurrence, which is the most important advantage of preserving sphincter function.

In conclusion, our study underscores the effectiveness of biliary stent placement in resolving small common bile duct stones while preserving sphincter function. Although this treatment method is expected to be effective in clearing approximately half of the common bile duct stones, complications such as cholecystitis, pancreatitis, and short‐term stone recurrence should be considered. For patients with acute cholangitis in whom physicians do not recommend EST, planned biliary stent placement could be attempted to treat small common bile duct stones.

## Data Availability

Data supporting the findings of this study are available from the corresponding author upon reasonable request.
